# Causes and intervention strategies for appearance anxiety: a critical review of recent advances

**DOI:** 10.3389/fpsyt.2026.1779181

**Published:** 2026-07-27

**Authors:** Junhao Wei, Yu Song, Mingjia Guo

**Affiliations:** 1 School of Basic Medical Sciences, Capital Medical University, Beijing, China; 2 Student Affairs Office, Capital Medical University, Beijing, China; 3 School of Marxism, Capital Medical University, Beijing, China

**Keywords:** appearance anxiety, body image-related psychopathology, causal mechanisms, intervention strategies, mental health

## Abstract

**Background:**

Appearance anxiety has increasingly been recognized as an important public mental health concern, characterized by persistent concerns regarding physical appearance and heightened sensitivity to social scrutiny. Visually oriented cultural norms, together with algorithm-driven social media environments, have been associated with appearance-related distress, particularly among adolescents and young adults, which may adversely affect psychosocial functioning.

**Objective:**

This study aims to develop a comprehensive biopsychosocial etiological model of appearance anxiety through a critical synthesis of empirical research published over the past decade (2015-2025). It also seeks to systematically assess the ecological validity and potential clinical applicability of existing intervention strategies.

**Methods:**

This narrative critical review synthesizes evidence from representative empirical and theoretical studies published primarily between 2015 and 2025. Relevant literature was identified through structured searches of major electronic databases, including PubMed, Web of Science, Scopus, and Google Scholar. Studies were selected based on their relevance to the conceptualization, etiological mechanisms, psychosocial consequences, and intervention strategies of appearance anxiety. Seminal studies published before 2015 were also included when necessary to provide theoretical and historical context for contemporary research.

**Results:**

The reviewed evidence suggests that appearance anxiety is associated with multiple interacting sociocultural, interpersonal, and individual factors: (1) Sociocultural factors: algorithmic content curation, coupled with the homogenized global beauty standards, may contribute to repeated exposure to “supernormal stimuli”; (2) Interpersonal factors: intergenerational transmission of parenting practices and rejection sensitivity within peer contexts form a social reinforcement mechanism for anxiety; (3) Individual factors: cognitive biases, such as upward social comparison, and personality traits, notably perfectionism, contribute to recursive, self-reinforcing cycles of negative feedback loop. Regarding interventions, while Cognitive Behavioral Therapy (CBT) is among the most extensively studied interventions and systemic prevention programs demonstrate notable advantages in accessibility and scalability.

**Conclusion:**

Appearance anxiety is a multidimensional psychological construct shaped by the interplay between sociocultural pressures and individual dispositions. Future research should transition from an exclusive emphasis on symptom relief to multidimensional ecological interventions, with particular attention to adaptive mechanisms in cross-cultural contexts and the long-term effectiveness of digital therapeutics.

## 1 Introduction

In contemporary visually oriented societies, appearance anxiety has increasingly been recognized as an important public mental health concern. In the present review, appearance anxiety is conceptualized as a dimensional psychological construct characterized by persistent concern, heightened self-monitoring, and anxiety-related emotional responses regarding one’s physical appearance. Rather than representing a formal psychiatric diagnosis, appearance anxiety exists along a continuum of severity and may range from normative appearance-related concerns to clinically relevant distress (1, 2).

Although appearance anxiety overlaps with several related constructs, these concepts are not interchangeable. Social Appearance Anxiety (SAA) primarily refers to fear of negative evaluation of one’s appearance in social situations, whereas appearance anxiety encompasses broader cognitive and emotional processes, including self-surveillance and appearance-related rumination that may occur even outside immediate social contexts. Similarly, appearance anxiety should be distinguished from body dissatisfaction, body image disturbance, Body Dysmorphic Disorder (BDD), eating-related psychopathology, and Social Anxiety Disorder, although substantial overlap may exist among these constructs (3).

Although adolescents and young adults are frequently discussed together in the appearance anxiety literature, important developmental differences should be acknowledged. During adolescence, appearance concerns are often closely linked to pubertal changes, identity formation, peer evaluation, and family influences. In contrast, young adulthood is characterized by greater autonomy and increased exposure to romantic, academic, and occupational evaluation. Nevertheless, both developmental stages involve heightened sensitivity to appearance-related feedback and therefore represent periods of increased vulnerability to appearance anxiety.

From a theoretical perspective, Social Comparison Theory and Self-Objectification Theory provide important frameworks for understanding appearance anxiety. Social Comparison Theory suggests that upward social comparison and resulting self-devaluation play a central role in the development of anxiety (4). Self-Objectification Theory further explains that when individuals internalize a third-person perspective as a mode of self-monitoring, body shame and the depletion of cognitive resources are likely to occur (5, 6).

Epidemiological data illustrate the gravity of this issue. Recent studies indicate a significant positive correlation between the frequency of social media usage and appearance anxiety, an association notably evident among adolescents and young adults (7). In samples of university students, a substantial majority report moderate to severe appearance anxiety, exhibiting complex gender binary differences. While females are more frequently represented in higher anxiety categories, recent studies have also reported substantial appearance-related concerns among males (8). These findings suggest that appearance anxiety may be shaped by the interaction of gender norms, cultural expectations, and broader social pressures.

Despite increasing empirical attention, existing research largely relies on single-dimensional correlational analyses and lacks an integrative examination of multiple etiological pathways. Accordingly, this review aims to synthesize empirical studies from the past decade to examine the sociocultural, family, and individual psychological factors contributing to appearance anxiety, and to critically evaluate the effectiveness of existing intervention strategies. By doing so, this review seeks to provide evidence-based insights for future clinical practice and public health policy.

## 2 Methods

### 2.1 Review design

This study was conducted as a narrative critical review aimed at synthesizing current evidence regarding the causes, psychosocial consequences, and intervention strategies of appearance anxiety. Rather than providing an exhaustive systematic synthesis, the review sought to integrate representative empirical findings and theoretical perspectives into a biopsychosocial framework.

### 2.2 Literature search strategy

Relevant literature was identified through structured searches of PubMed, Web of Science, Scopus, and Google Scholar. The search focused primarily on studies published between 2015 and 2025 using keywords including “appearance anxiety”, “social appearance anxiety”, “body image”, “body dissatisfaction”, “body dysmorphic disorder”, “social media”, “self-objectification”, “cognitive behavioral therapy”, “mindfulness”, and “digital intervention”. Reference lists of relevant reviews and influential studies were also screened manually. Although the primary focus was on literature published between 2015 and 2025, several seminal studies published before 2015 were retained because they provided important theoretical foundations and historical context for contemporary research.

### 2.3 Inclusion and exclusion criteria

Studies were included if they examined appearance anxiety or closely related body image constructs, investigated etiological mechanisms or intervention strategies, and were published in peer-reviewed journals. Empirical studies, systematic reviews, meta-analyses, and influential theoretical papers were considered. Editorials, conference abstracts, non-peer-reviewed sources, and studies unrelated to appearance anxiety were excluded.

### 2.4 Evidence synthesis

The included literature was synthesized thematically according to a biopsychosocial framework encompassing sociocultural, interpersonal, and individual psychological factors, as well as intervention approaches. Greater interpretive weight was given to systematic reviews, meta-analyses, randomized controlled trials, and longitudinal studies, while findings from cross-sectional studies were interpreted with appropriate caution regarding causal inference. In addition, the geographical distribution of the reviewed studies was examined to provide greater transparency regarding the cultural contexts represented in the evidence base.

After verification of the final reference list, 71 studies were included in this narrative critical review. Among these studies, approximately 25.4% were conducted in Asia, 29.6% in Europe, 19.7% in North America, 8.5% in Oceania, and 1.4% in Latin America, while 15.5% involved multi-country or international samples. Although the evidence base was geographically diverse, studies from Asia, Europe, and North America accounted for the majority of the literature, highlighting the need for broader representation from underrepresented regions in future appearance anxiety research.

Because this review was conducted as a narrative critical review rather than a formal systematic review, evidence strength was evaluated using a narrative appraisal approach rather than the GRADE framework. The evidence classifications presented in **Tables 1**, **2** were based on four complementary considerations: (1) the consistency of findings across studies; (2) the methodological quality of the available evidence, with greater weight given to systematic reviews, meta-analyses, randomized controlled trials, longitudinal studies, and well-designed observational studies; (3) the quantity of available evidence; and (4) the direct relevance of the evidence to appearance anxiety. Accordingly, Strong indicates consistent evidence from multiple high-quality or complementary studies, Moderate indicates evidence supported by several studies but with methodological limitations or less consistent findings, and Emerging indicates preliminary, indirect, or limited evidence requiring further validation.

**Table 1:** Summary of major etiological factors associated with appearance anxiety.

Domain	Factor	Representative evidence	Proposed mechanism	Strength of evidence
Sociocultural	Social media exposure	Social media use is consistently associated with higher appearance anxiety and body image concerns among adolescents and young adults (7, 25, 27).	Upward social comparison and appearance-focused self-evaluation.	Strong
Sociocultural	Social comparison and appearance ideals	Exposure to idealized appearance content may promote internalization of unrealistic beauty standards (13, 25).	Increased self-discrepancy and body dissatisfaction.	Strong
Sociocultural	Consumer culture and appearance norms	Global body image concern and cultural/gender appearance norms (15, 29)	Appearance becomes an important source of self-worth and social evaluation.	Moderate
Family	Parental criticism and appearance-related feedback	Parental dissatisfaction and critical comments predict greater appearance-related concerns (30–32).	Internalization of negative appearance evaluations.	Moderate
Family	Attachment insecurity	Insecure attachment is associated with greater body dissatisfaction and appearance-related vulnerability (33).	Increased need for external validation and fear of rejection.	Moderate
Peer	Peer rejection and appearance teasing	Appearance-related rejection and teasing are associated with body image concerns and social anxiety (34, 35).	Heightened sensitivity to social evaluation.	Moderate
Peer	Cyberbullying	Appearance-related cyberbullying predicts appearance dissatisfaction and appearance-modification intentions (36).	Appearance-based victimization and self-monitoring.	Moderate
Individual	Perfectionism	Perfectionism is a shared vulnerability factor for appearance anxiety, social anxiety, and eating-related symptoms (38).	Unrealistic appearance standards and self-criticism.	Strong
Individual	Low self-esteem	Lower self-esteem is associated with greater appearance-related distress (39, 44).	Negative self-evaluation and dependence on external approval.	Moderate
Individual	Emotion regulation difficulties	Difficulties in emotional regulation may exacerbate appearance-related distress (39, 44).	Reduced coping capacity when facing appearance-related stressors.	Moderate

**Table 2:** Summary of representative intervention approaches for appearance anxiety and related outcomes.

Intervention approach	Representative study	Evidence type	Target population	Main outcomes	Main findings	Evidence level
Cognitive Behavioral Therapy (CBT)	Wheaton et al., 2025 (54); Kerry et al., 2024 (55); Chopra et al., 2024 (56)	Clinical studies/RCTs	BDD and body image-related populations	Appearance-related distress; body image	Consistent symptom improvement across clinically relevant populations.	Strong
Mindfulness-Based CBT	Kerry et al., 2024 (55)	Pilot RCT	Individuals with BDD	Appearance preoccupation; emotional distress	Preliminary evidence suggests beneficial effects on appearance-related symptoms.	Moderate
Internet-Based CBT (iCBT)	Andersson & Titov, 2014 (57); Etzelmueller et al., 2020 (58)	Systematic review and meta-analysis	Anxiety and depression populations	Anxiety symptoms; psychological well-being	Effective and scalable digital intervention model; evidence remains indirect for appearance anxiety.	Moderate–Strong
Digital Body Image Interventions	Matheson et al., 2023 (14); Mahon & Seekis, 2022 (17)	RCT; systematic review	Adolescents and young adults	Body image; mental health outcomes	Demonstrated positive effects on body image and psychological well-being.	Moderate
Media Literacy Programs	Kurz et al., 2022 (69)	Systematic review and meta-analysis	Adolescents and students	Body image; appearance ideals	Effective preventive strategy for reducing harmful media influences.	Strong
Self-Compassion-Based Approaches	Gao et al., 2023 (64); Fan et al., 2022 (70)	Observational and intervention-related evidence	Individuals with appearance-related concerns	Appearance anxiety; social anxiety	Self-compassion may buffer appearance-related distress and enhance resilience.	Moderate
Drama Therapy	Berghs et al., 2022 (51)	Systematic review	Children and adolescents	Psychosocial functioning	Promising psychosocial benefits, but direct appearance anxiety evidence is limited.	Emerging
Music Therapy	de Witte et al., 2022 (52)	Systematic review and meta-analysis	Stress-related populations	Stress reduction	Potential supportive role; evidence remains indirect.	Emerging
Dance-Based Interventions	Liu et al., 2025 (53)	Systematic review	Adults	Social physique anxiety; body self-esteem	Improved body self-esteem and reduced physique-related anxiety.	Emerging–Moderate
AI-Assisted Assessment and Intervention	Milasan & Scott-Purdy, 2025 (59); Monteith et al., 2022 (60)	Review and conceptual evidence	Digital mental health settings	Assessment; personalization	Promising future direction, but appearance anxiety-specific evidence remains limited.	Emerging/Theoretical

## 3 Conceptual evolution and theoretical perspective on appearance anxiety

### 3.1 Sociocultural influence on appearance anxiety

The conceptualization of appearance anxiety has evolved from a unidimensional construct to a multidimensional one, and from descriptive accounts of symptoms to greater attention to underlying psychological mechanisms. Early research often equated appearance anxiety with body image dissatisfaction, overlooking its anticipatory and socially evaluative nature (9). Meta-analytic evidence has identified gender differences in the association between body appreciation and body mass index, suggesting that appearance anxiety involves more complex psychological processes than previously assumed (9).

Contemporary studies increasingly conceptualize appearance anxiety as a multidimensional construct encompassing several components: (1) a cognitive dimension, involving negative evaluations and catastrophizing thoughts about one’s appearance; (2) an emotional dimension, including anxiety, shame, and fear related to appearance; (3) a behavioral dimension, characterized by avoidance, concealment, and excessive grooming behaviors; and (4) a physiological dimension, reflected in heightened arousal when individuals encounter appearance-related situations. Further research has identified sequential mediation pathways between appearance anxiety and social anxiety, with self-efficacy and self-esteem playing key roles (10).

### 3.2 Cultural adaptability of measurement tools

Existing measurement instruments for appearance anxiety exhibit notable cultural limitations. Many scales developed in Western contexts are grounded in individualistic cultural assumptions that emphasize personal autonomy and independent self-evaluation. Although some visual-perceptual assessment methods have demonstrated cross-cultural validity within Western populations, their applicability in Asian cultural settings remains insufficiently examined (11).

In some East Asian contexts, collectivist-oriented values and concerns regarding social image or “face” may confer distinct social meanings on appearance anxiety. Cross-cultural research has revealed significant differences in perceptions of female facial appearance across ethnic groups, reflecting not only aesthetic preferences but also deeply embedded cultural values (12). In social contexts shaped by Confucian traditions, appearance is often understood not merely as an individual attribute but as a reflection of family honor, which may intensify appearance-related anxiety at the individual level.

### 3.3 Integration and critique of theoretical models

#### 3.3.1 Sociocultural perspectives

Experimental studies on fitspiration content suggest that even ostensibly positive body-related messages may exacerbate appearance anxiety by reinforcing ideals of the “perfect” body (13). These findings challenge the assumption that positive messaging necessarily produces positive psychological outcomes and underscore the complexity of sociocultural influences on appearance-related distress.

Feminist theories provide a power-oriented framework for understanding appearance anxiety (14). In patriarchal social structures, the “beauty myth” links women’s social value to physical appearance, encouraging sustained investment of time, energy, and economic resources in appearance management, thereby reinforcing existing gender power relations (15, 16). Although digital interventions targeting adolescents and young women have shown some effectiveness, existing studies indicate that such approaches rarely address the broader structural factors that contribute to appearance anxiety (17–19).

#### 3.3.2 Evolutionary psychology perspective

From an evolutionary psychology perspective, attention to physical appearance may have served adaptive functions related to mate selection and access to social resources (20–22). However, contemporary technological practices, such as digital image manipulation and cosmetic surgery, have produced “supernormal stimuli” that were absent in evolutionary history. As a result, psychological mechanisms shaped by natural selection may become maladaptive under modern conditions (23).

### 3.4 Distinction from related constructs

Consistent with the definition adopted in this review, appearance anxiety is conceptualized as a dimensional and transdiagnostic psychological phenomenon rather than a formal psychiatric diagnosis. Although it shares features with several body image-related and anxiety-related constructs, important conceptual distinctions should be maintained. Appearance anxiety should be distinguished from several related but conceptually distinct conditions. Body Dysmorphic Disorder (BDD) is a psychiatric condition characterized by pathological preoccupation with imagined or minor defects in physical appearance. Systematic reviews indicate that body image disturbances in BDD and muscle dysmorphia are multidimensional, encompassing cognitive, emotional, perceptual, and behavioral components (20). Unlike appearance anxiety as conceptualized in this review, BDD involves clinically significant distress and functional impairment.

**Figure 1:**
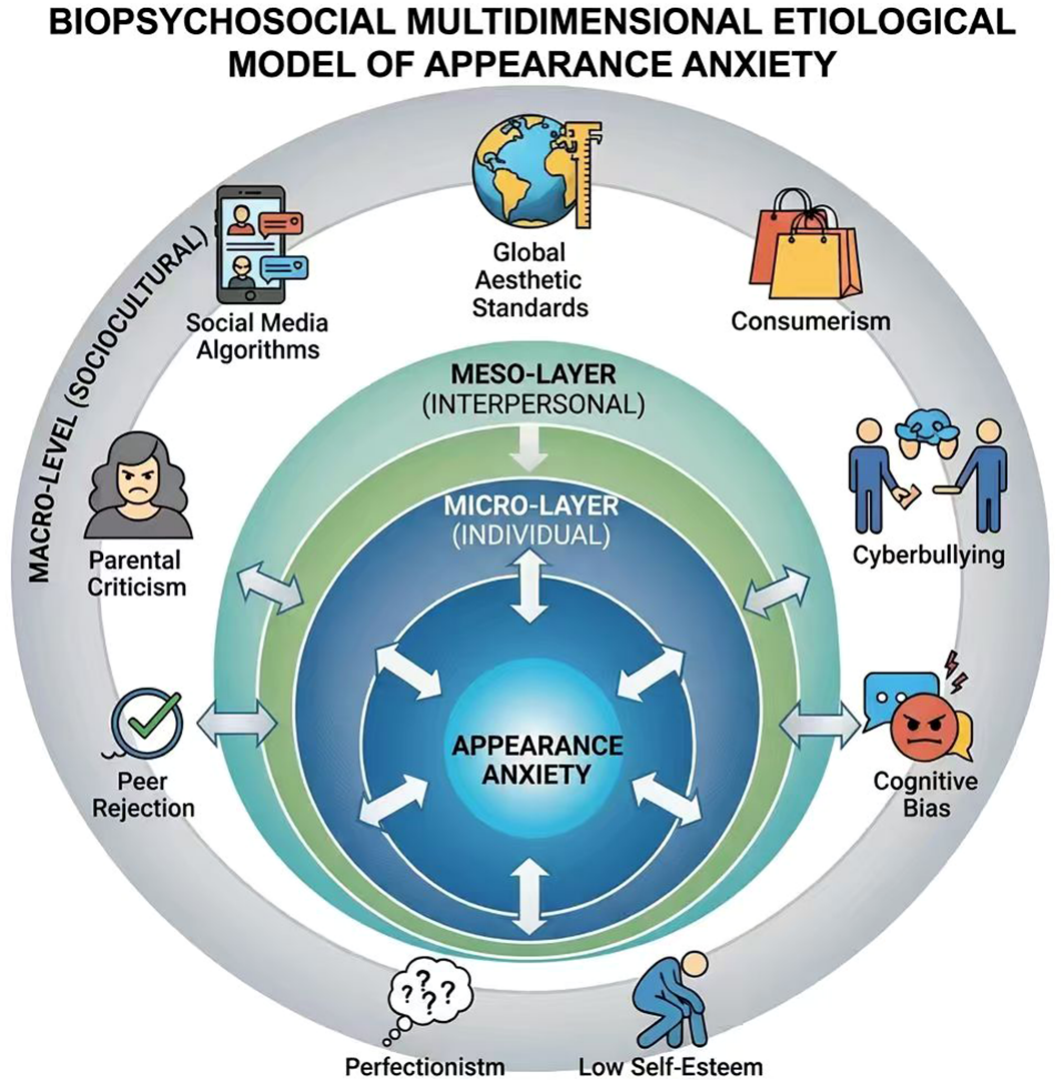
Conceptual biopsychosocial framework of appearance anxiety. This figure presents a heuristic conceptual synthesis derived from the literature reviewed in this article. The proposed pathways organize recurrent findings into sociocultural, interpersonal, and individual domains that may contribute to appearance anxiety and are intended to illustrate potential interactions among these domains rather than validated causal relationships. The strength of evidence supporting individual pathways varies across domains, with some relationships supported by longitudinal and experimental studies, whereas others are primarily informed by cross-sectional or theoretical evidence.

**Figure 1** represents a conceptual synthesis of the recurring factors identified across the reviewed literature rather than a formally validated causal model. The framework was developed by organizing evidence into three interacting domains: sociocultural influences (e.g., social media environments, appearance norms, and consumer culture), interpersonal influences (e.g., parental criticism, peer rejection, and cyberbullying), and individual psychological characteristics (e.g., cognitive biases, perfectionism, and self-esteem). Although many of these associations are supported by empirical studies, the strength of evidence varies considerably. Relationships involving social media use, social comparison, and body image concerns are supported by longitudinal and experimental research, whereas broader sociocultural mechanisms remain more strongly informed by theoretical and cross-sectional evidence (**Figure 1**). Accordingly, the pathways illustrated in **Figure 1** should be interpreted as heuristic representations of potential relationships rather than as evidence of established causal mechanisms. The directional arrows are intended to facilitate conceptual understanding and do not imply deterministic or universally applicable causal pathways.

## 4 Etiological mechanisms of appearance anxiety

### 4.1 Sociocultural factors

#### 4.1.1 Impact of digital media environment

The mechanisms through which social media use has been associated with appearance anxiety extend well beyond a simple exposure-effect model. Empirical research indicates that multiple pathways link social media use to appearance anxiety among adolescents and young adults (7). In particular, passive browsing has been shown to elicit stronger upward social comparison and appearance dissatisfaction than active content creation or posting. Systematic reviews further suggest that the impact of social media on body image is jointly shaped by the intensity and type of use, as well as individual characteristics (23).

The role of algorithmic recommendation systems cannot be overlooked. Studies grounded in self-objectification theory indicate that sociocultural pressures influence appearance anxiety through mediation processes involving the self-system (24). By reinforcing users’ existing preferences, platform algorithms may generate “filter bubbles” that repeatedly expose individuals to idealized body images. Longitudinal research employing random intercept cross-lagged panel models has further demonstrated reciprocal temporal associations between adolescents’ engagement with visually oriented platforms and body-image-related constructs (25).

In addition, the relationship between information overload and selective exposure warrants attention. Evidence suggests a complex relationship between information hoarding and selective exposure, wherein identity bubble reinforcement and intolerance of uncertainty play regulatory roles (26). This mechanism may trap individuals in an “aesthetic cocoon,” constantly reinforcing uniform aesthetic standards.

#### 4.1.2 Cultural values and aesthetic hegemony

Aesthetic standards across cultural contexts are not inherently objective or natural but are socially constructed expressions of underlying value systems and power relations. In some societies characterized by high power distance or collectivist orientations, dominant appearance norms are often systematically reinforced and reproduced through educational institutions, mainstream media, and social norms. Within such environments, deviations from normative aesthetic ideals may carry heightened social consequences, thereby shaping culturally specific associations between social media engagement and appearance anxiety (27).

From a broader historical perspective, shifts in aesthetic ideals have frequently paralleled transformations in social power structures (28). In the context of globalization, Western-centric beauty standards have gained disproportionate global influence through cultural industries and consumer culture, contributing to what has been described as a form of aesthetic hegemony in many non-Western societies. Importantly, this process does not operate as a unidirectional cultural imposition. Local cultures actively resist, reinterpret, and hybridize global aesthetic influences, resulting in a dynamic and heterogeneous contemporary aesthetic landscape (29).

Tensions are particularly evident at the intersection of traditional values and rapidly evolving media environments. In transitional societies, collectivist norms emphasizing conformity, social reputation, and “face” coexist uneasily with media discourses that advocate individuality, visibility, and self-presentation. This cultural dissonance may intensify identity-related stress among young people and situate appearance anxiety at the intersection of individual psychological vulnerability and broader sociocultural change.

#### 4.1.3 Integrative mechanisms of sociocultural influence

Taken together, sociocultural factors appear to shape appearance anxiety primarily through three interrelated mechanisms. First, visually oriented media environments increase exposure to idealized and often digitally modified appearance standards, which may intensify upward social comparison and perceived discrepancies between the actual and ideal self. Second, algorithmic recommendation systems may amplify this process by repeatedly presenting appearance-focused content to users who have previously engaged with similar material, thereby reinforcing self-objectification and appearance monitoring. Third, cultural values and gendered aesthetic norms influence how appearance is socially evaluated, determining whether physical appearance is interpreted merely as a personal attribute or as a marker of social value, discipline, desirability, or family and group reputation.

However, the strength of evidence differs across these mechanisms. The association between social media use, appearance comparison, and body image concerns is supported by a relatively large body of cross-sectional, longitudinal, experimental, and review evidence. By contrast, claims regarding algorithmic curation and aesthetic hegemony remain more theoretically driven and require further empirical testing, particularly in non-Western cultural contexts. Therefore, sociocultural influences should be understood not as direct deterministic causes of appearance anxiety, but as distal contextual pressures that interact with interpersonal and individual vulnerabilities to increase appearance-related distress.

### 4.2 Family environment factors

#### 4.2.1 Intergenerational transmission of parental influence

Intergenerational transmission within the family system may represent an important pathway in the development of appearance anxiety. Empirical evidence indicates that parents’ body dissatisfaction not only directly predicts body image distress in children-particularly between the ages of 9 and 11-but is also transmitted across generations through mediating pathways involving restrictive eating behaviors and excessive concern with offspring’s weight (30). This process extends beyond simple behavioral imitation and reflects a systematic internalization of body-related attitudes, aesthetic beliefs, and emotional response patterns.

Importantly, this transmission is not deterministic. Parents’ psychological resilience plays a key regulatory role in shaping intergenerational outcomes. Research suggests that adaptive coping strategies and higher levels of parental acceptance under situational stress can effectively buffer the intergenerational transmission of negative body image (31). These findings indicate that mature emotion regulation abilities and psychological resilience among parents may function as protective mechanisms within the family system, interrupting the progression of appearance-related anxiety.

In contrast, invasive parenting styles substantially exacerbate pathological risk. Parental overcontrol has been consistently associated with greater severity of dysmorphic concerns in children (32). Within collectivist cultural contexts, such boundary-eroding practices-often framed as expressions of “care”-may restrict children’s psychological space for developing autonomous aesthetic values and self-acceptance. As a result, individuals may be compelled to internalize external evaluations as standards for self-monitoring, thereby reinforcing cognitive schemas associated with appearance anxiety.

#### 4.2.2 Influence of attachment relationships

Attachment dynamics further shape vulnerability to appearance-related distress. Studies indicate that parental bonding influences body dissatisfaction through indirect pathways involving attachment anxiety and the internalization of media ideals (33). Individuals with insecure attachment patterns, particularly anxious attachment, tend to rely more heavily on external evaluation to maintain self-worth, rendering them more susceptible to appearance-related pressures.

#### 4.2.3 Family environment as a proximal social mechanism

Overall, family-related influences may contribute to appearance anxiety by shaping the early emotional and cognitive framework through which individuals interpret their bodies and appearance. Parental body dissatisfaction, weight-related comments, overcontrol, and conditional acceptance may increase children’s sensitivity to external evaluation and encourage the internalization of appearance-based standards. Attachment insecurity may further amplify this vulnerability by making self-worth more dependent on external approval and perceived social acceptance.

Importantly, family influences should not be interpreted as isolated or deterministic causes. Rather, they may operate as proximal social mechanisms through which broader sociocultural appearance norms are transmitted, reinforced, or buffered within everyday interpersonal relationships. Supportive parenting, parental acceptance, and adaptive emotion regulation may function as protective factors, whereas intrusive control and appearance-focused criticism may increase vulnerability. This distinction is important because it shifts the interpretation from a simple “parental influence” model to a more dynamic framework in which family environments mediate and moderate the impact of wider cultural pressures on appearance anxiety.

### 4.3 Peer environment and social interaction

Research has identified gender differences in the relationship between appearance-related teasing and mental health outcomes, with appearance-based rejection sensitivity and dysmorphic concerns functioning as key mediating mechanisms (34, 35). Even seemingly benign “jokes” during critical developmental periods may exert enduring psychological effects.

Cyberbullying may represent an additional pathway for appearance-related harm. Empirical evidence indicates that appearance-focused cyberbullying is significantly associated with an increased desire among adolescent females to alter their physical appearance (36). The anonymity and permanence of online environments further intensify the impact of such experiences, rendering the resulting psychological harm more difficult to prevent and remediate.

Peer-related experiences may serve as an important pathway through which sociocultural appearance ideals are translated into individual appearance anxiety. Through peer comparison, appearance-based teasing, rejection sensitivity, and cyberbullying, cultural beauty standards become personally relevant and socially consequential. These experiences may increase vigilance toward perceived appearance flaws and reinforce the belief that physical appearance influences social acceptance and interpersonal value. Such mechanisms may be particularly salient during adolescence and emerging adulthood, when peer approval and social belonging assume heightened developmental importance. However, because much of the available evidence is correlational, the directionality of these associations remains unclear, and future longitudinal research is needed to clarify the reciprocal relationship between peer experiences and appearance anxiety.

Such mechanisms may be particularly salient during adolescence and emerging adulthood, although the underlying developmental processes may differ. Peer acceptance, identity formation, and pubertal changes may play a more prominent role during adolescence, whereas romantic relationships, occupational evaluation, and social independence may become increasingly important during young adulthood.

### 4.4 Individual psychological factors

#### 4.4.1 Personality traits and cognitive styles

Cross-temporal meta-analyses have documented an upward trend in social anxiety among university students over recent decades (37). This temporal shift likely reflects broader systemic changes in social structure, cultural values, and lifestyle. Further investigation into the generative mechanisms of appearance anxiety highlights perfectionism, levels of self-esteem, and maladaptive cognitive biases as key individual-level factors (38).

Negative physical self-concept has been shown to mediate the relationship between emotion regulation strategies and social anxiety (39). Individuals who predominantly rely on avoidance or emotional suppression are more likely to develop appearance-related anxiety. Studies conducted in university populations further indicate that factors contributing to social anxiety include introversion, low self-evaluation, and insufficient social support (40).

#### 4.4.2 Self-efficacy and emotion regulation

The availability of individual psychological resources plays a pivotal role in determining whether appearance-related concerns escalate from latent risk into overt symptoms. Empirical evidence suggests that emotion regulation capacity-particularly the effective use of cognitive reappraisal strategies-serves as an important protective factor, buffering the negative impact of appearance anxiety on quality of life (41). These findings indicate that anxiety severity is shaped less by perceived appearance flaws themselves than by individuals’ cognitive and emotional processing of appearance-related experiences.

From a cognitive-dynamic perspective, self-efficacy occupies a central mediating position. Drawing on social cognitive theory (42), low self-efficacy not only directly increases vulnerability to anxiety but also exerts indirect effects by activating negative self-schemas, thereby generating a self-reinforcing cycle of low efficacy, maladaptive cognition, and heightened anxiety (43).

Furthermore, network analyses in psychopathology suggest that the boundary between appearance anxiety and Social Anxiety Disorder (SAD) is highly permeable. Although the core pathology of SAD centers on a generalized fear of social evaluation, fear of negative evaluation has been identified as a key “bridge symptom” linking these two conditions (44). This observation implies that interventions targeting this shared symptom may yield transdiagnostic therapeutic benefits.

### 4.5 Bidirectional relationship between dietary behavior and appearance anxiety

Available evidence suggests a complex bidirectional association between appearance anxiety and disordered eating, a relationship further supported by findings from symptom network analyses. Research indicates that “fear of weight gain” and “excessive concern with body shape” function as core symptom nodes linking anxiety-related affect with pathological eating behaviors. These central nodes play a critical role in maintaining the stability of the comorbidity network (45).

From a behavioral perspective, emotional eating often emerges as a maladaptive compensatory strategy. Although it may provide short-term relief from anxiety through dopaminergic reward mechanisms, over time this unregulated eating pattern exacerbates body dissatisfaction, thereby reinforcing a self-perpetuating negative feedback loop between anxiety and eating pathology (46).

Digital media environments further intensify this process. The widespread circulation of thinspiration and fitspiration content on social media promotes unrealistic body ideals (47). Analyses based on social comparison theory demonstrate that sustained exposure to such idealized imagery encourages restrictive eating behaviors among young women as a means of reducing perceived discrepancies between actual and idealized bodies (48). Notably, this form of body discipline exhibits pronounced gender differentiation: longitudinal evidence suggests that appearance-related anxiety in adult males is more strongly oriented toward muscularity ideals, whereas females are disproportionately constrained by thinness norms, reflecting the differential shaping of dietary pathology by sociocultural expectations (49).

Taken together, the etiological mechanisms reviewed in this section point to a multi-level framework in which sociocultural, family, peer-related, and individual factors jointly contribute to appearance anxiety. **Table 1** provides a structured summary of these major cross-domain etiological factors reviewed throughout Section 4.

## 5 Intervention strategies and effectiveness evaluation

### 5.1 Psychotherapeutic interventions

#### 5.1.1 Traditional psychotherapeutic approaches

**Figure 2:**
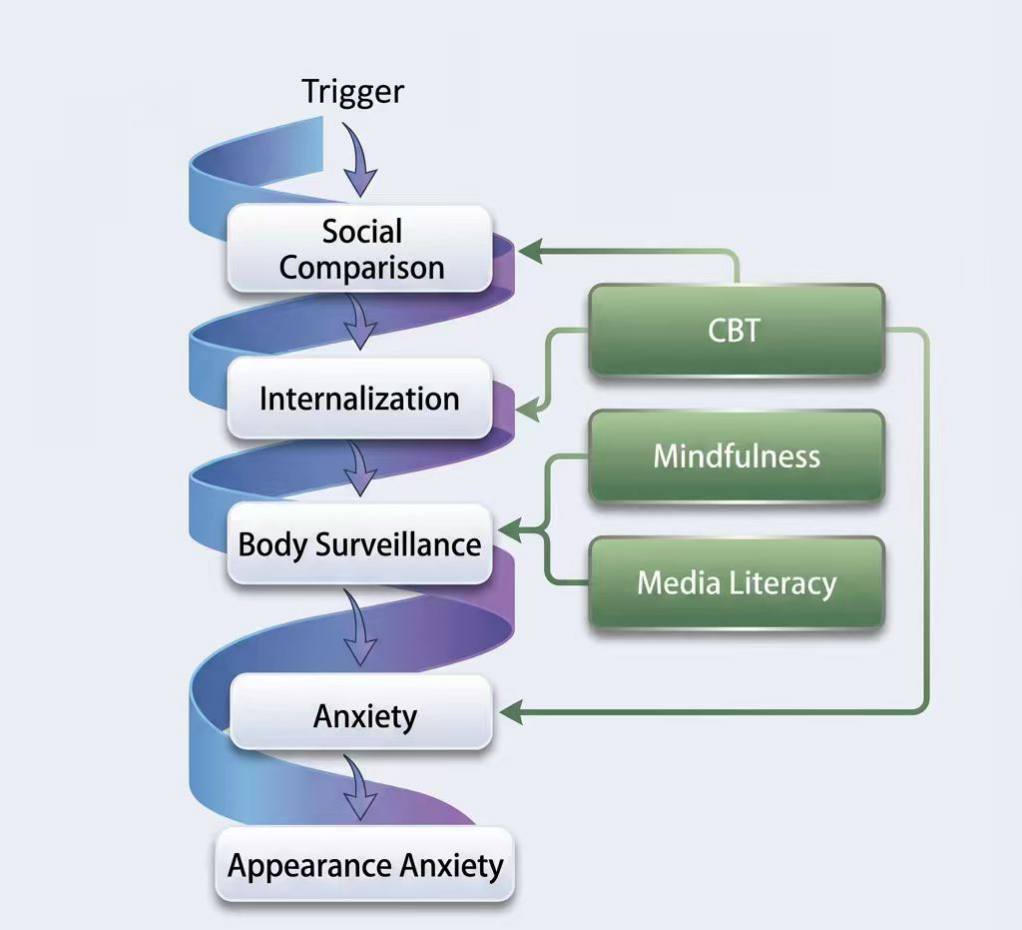
Heuristic cognitive-behavioral pathway and potential intervention targets for appearance anxiety. This figure summarizes commonly proposed psychological mechanisms discussed in the reviewed literature and maps intervention strategies onto different stages of the pathway. The sequence should be interpreted as a heuristic framework rather than a definitive causal model, as the supporting evidence varies in methodological strength across different components.

**Figure 2** summarizes a heuristic cognitive-behavioral pathway derived from the theoretical and empirical literature reviewed in this article. The sequence of social comparison, internalization, body surveillance, and anxiety is intended to illustrate commonly proposed psychological mechanisms rather than a universally established causal progression. Evidence supporting these mechanisms varies across stages and includes experimental studies of social comparison processes, longitudinal studies of body image development, cross-sectional investigations of self-objectification, and broader theoretical models (**Figure 2**).

Traditional psychological intervention models are increasingly challenged to move beyond a “one-size-fits-all” paradigm toward “precise medicine.” Recent studies indicate that interventions for depression and anxiety in neurodiverse populations must be tailored to their specific cognitive processing traits, underscoring the importance of accounting for neurobiological diversity in interventions for appearance anxiety (50).

Meanwhile, non-verbal expressive arts therapies offer alternative pathways to overcome the limitations of linguistic cognition. Systematic reviews confirm that drama therapy provides a safe psychological rehearsal space for adolescents through role-playing and projection techniques, thereby facilitating the reconstruction of psychosocial functioning (51). Meta-analyses further quantify the stress-reducing effects of music therapy, noting that rhythmic and harmonic elements can directly modulate limbic system arousal and promote emotional release (52). Other studies on embodied cognition have shown that dance interventions can significantly lower social physique anxiety and boost body self-esteem in adults by re-establishing a positive embodied connection between the individual and the body through movement (53). Although these expressive and embodied intervention approaches have demonstrated promising effects on body image, stress reduction, and psychosocial functioning, their evidence base remains more limited. Moreover, much of the available evidence originates from adjacent populations and related body image outcomes rather than appearance anxiety-specific trials.

#### 5.1.2 Cognitive behavioral therapy and its variants

Cognitive behavioral therapy (CBT) is among the most extensively studied intervention approaches for appearance-related distress and related body image psychopathology. However, a considerable proportion of the available evidence has been derived from clinically related conditions, particularly obsessive-compulsive disorder (OCD), body dysmorphic disorder (BDD), and other body image-related disorders, rather than appearance anxiety specifically. For example, studies in patients with OCD have shown that CBT not only alleviates clinical symptoms but also enhances neurocognitive performance, suggesting that psychological interventions may exert their effects by reshaping underlying neural circuits. Although these findings provide indirect support for the potential value of CBT in appearance anxiety, direct evidence in appearance anxiety populations remains comparatively limited (54). Among the intervention approaches reviewed, CBT and its variants currently possess the strongest empirical support, with evidence derived primarily from randomized controlled trials and studies conducted in clinically relevant populations.

With the rise of so-called “third-wave” therapies, the integration of acceptance- and mindfulness-based elements has become an important trend. Randomized controlled trials have shown that web-based, therapist-guided mindfulness-based CBT demonstrates superior efficacy in the treatment of BDD, with mindfulness training effectively interrupting maladaptive cycles of ruminative thinking and avoidance behaviors (55). However, because much of the available evidence originates from BDD and related body image disorders, the extent to which these findings generalize to broader appearance anxiety populations remains uncertain. In addition, for specific clinical populations with physical impairments-such as patients with facial cancer-adapted CBT protocols have been shown to significantly improve body image outcomes, supporting the ecological validity of targeted interventions in specialized clinical contexts (56).

### 5.2 Digital transformation: computational psychiatry and AI empowerment

#### 5.2.1 Accessibility and algorithmic optimization of internet interventions

The rise of digital medicine has substantially expanded the reach of psychological interventions. Studies indicate that internet-based interventions have significant advantages in enhancing accessibility and reducing stigmatization. However, the absence of a sustained therapeutic alliance remains a central limitation (57). Meta-analytic evidence further demonstrates that integrating internet-based cognitive behavioral therapy (iCBT) into routine care yields moderate to large effect sizes for anxiety and depression, supporting its role as a foundational component within stepped-care models. However, these findings are primarily derived from anxiety and depression populations rather than individuals with appearance anxiety. Therefore, although they provide supportive evidence for the feasibility and scalability of digital CBT-based interventions, their applicability to appearance anxiety should be interpreted cautiously until more disorder-specific studies become available (58). Compared with emerging digital approaches, iCBT is supported by a relatively stronger evidence base, including systematic reviews and meta-analyses conducted across anxiety and depression populations.

#### 5.2.2 Artificial intelligence as a future direction for digital mental health

The rapid development of artificial intelligence (AI) has generated growing interest in its potential applications within digital mental health. In the context of appearance anxiety, AI-assisted technologies may offer new opportunities for early identification, personalized assessment, and adaptive intervention delivery, particularly among adolescents and young adults who frequently engage with digital platforms. Emerging research in digital mental health suggests that AI-based systems may help tailor intervention content according to individual symptom profiles, behavioral patterns, and treatment responses (59). Such approaches have shown promise in related areas, including anxiety, depression, body image concerns, and eating-related psychopathology. However, direct evidence supporting the effectiveness of AI-assisted interventions specifically for appearance anxiety remains limited.

Several challenges also warrant consideration, including concerns regarding privacy protection, algorithmic bias, transparency, and ethical use of personal data. Furthermore, most existing applications have been evaluated within broader mental health contexts rather than appearance anxiety itself. Therefore, AI-based approaches should currently be regarded as promising future research directions rather than established evidence-based interventions for appearance anxiety (60). Future studies should investigate the feasibility, acceptability, and long-term effectiveness of AI-assisted assessment and intervention strategies while ensuring appropriate ethical safeguards and clinical oversight.

### 5.3 Preventive and group interventions

For university students and youth populations, computer- and internet-based intervention models have become preferred strategies for mental health promotion in resource-constrained contexts due to their scalability, accessibility, and potential for broad population reach. Meta-analytic evidence suggests that computer- and internet-based interventions may reduce anxiety symptoms and improve psychological well-being in selected populations. Although these findings support the broader potential of digital mental health interventions, most studies have been conducted in anxiety, depression, or general mental health populations rather than individuals with appearance anxiety. Consequently, the available evidence should be regarded as indirect when considering its application to appearance anxiety, highlighting the need for disorder-specific intervention studies (61, 62). With respect to intervention content, the well-documented association between media exposure and body image distress has positioned media literacy education as a core preventive strategy aimed at deconstructing idealized body standards perpetuated by media environments (63). However, most evidence supporting digital preventive interventions originates from studies of anxiety, depression, and general mental health outcomes rather than appearance anxiety specifically. Therefore, caution is warranted when extrapolating these findings to appearance anxiety populations.

Beyond externally delivered interventions, the mobilization of intra-individual psychological resources is equally critical. Self-compassion has been identified as a key mediating factor that interrupts the progression of appearance anxiety into social anxiety (64), suggesting that intervention goals should extend beyond symptom reduction toward the cultivation of positive psychological capacities. Moreover, interventions targeting the social media environment must account for the complex bidirectional relationship between digital media use and mental health (65). Early platform-specific studies have demonstrated that social comparison mechanisms within digital environments exert a substantial erosive effect on the body image of adolescent females, underscoring the need for preventive strategies that are finely tuned to platform-specific affordances (66).

### 5.4 Cultural adaptive interventions

The ecological validity of intervention protocols depends on their adaptability to sociocultural and individual differences. First, gender heterogeneity cannot be overlooked: emotional eating has been shown to mediate the relationship between anxiety and body image in a gender-specific manner, necessitating the integration of gender-sensitive perspectives into intervention design (67). Second, cross-cultural transplantation presents significant methodological challenges. The introduction of body image assessment tools and intervention manuals requires rigorous evaluation of conceptual equivalence, measurement invariance, and construct validity, rather than mere linguistic translation. In addition, interventions should consider culturally specific meanings of appearance, gender norms, and social expectations that may influence how appearance-related concerns are experienced and expressed across different populations (68).

At the implementation level, school-based intervention programs may represent a feasible approach for improving adolescents’ body image and media literacy within educational settings (69). However, the successful implementation of such programs may depend on local educational resources, socioeconomic conditions, cultural expectations, and the acceptability of intervention content within specific communities. More importantly, interventions must prioritize the prevention of severe downstream consequences. Given the documented association between body dissatisfaction and suicidal ideation, self-compassion has been shown to effectively regulate this high-risk pathway (70). This finding not only underscores the potential clinical severity of appearance anxiety but also reinforces the protective value of emotion regulation training centered on self-compassion in crisis prevention.

Taken together, the intervention strategies reviewed in Section 5 reflect a multi-dimensional and increasingly integrated framework encompassing psychotherapeutic interventions, digital and AI-driven mental health innovations, preventive and group-based programs, and culturally adaptive approaches. Despite differences in methodological grounding and evidence strength, these approaches collectively from a coherent intervention landscape. **Table 2** provides a structured synthesis of representative intervention approaches and their reported effectiveness across different populations and study designs.

## 6 Psychosocial consequences of appearance anxiety

### 6.1 Mental health impact

Appearance anxiety is not merely an isolated psychological concern but frequently co-occurs with depressive, anxiety-related, and eating-related symptoms. Empirical evidence indicates that the prevalence of depressive symptoms among individuals with elevated appearance anxiety is significantly higher than that observed in the general population (44). Such comorbidity may emerge through multiple interacting mechanisms, including ruminative cognitive processes, behavioral patterns of social withdrawal, and dysregulation of stress-response systems at the neurobiological level (71).

Impairment in social functioning represents another major consequence of appearance anxiety. Affected individuals may avoid social situations involving perceived “self-display,” such as parties, romantic encounters, or job interviews (40). This avoidance is maintained through negative reinforcement-providing short-term anxiety relief while progressively exacerbating social isolation and functional skill degradation over time. Studies conducted during the COVID-19 pandemic further revealed that the widespread reliance on video conferencing imposed additional psychological strain on individuals with appearance anxiety; experiences of “Zoom fatigue” were partly attributable to heightened and continuous self-monitoring (31).

### 6.2 Academic and occupational impact

The influence of appearance anxiety on academic and occupational trajectories is frequently underestimated. Research suggests that students with high levels of appearance anxiety are more likely to avoid or miss coursework involving public speaking, thereby undermining academic performance (38). In occupational contexts, individuals may self-select out of positions that require visible “image display,” which can constrain career choices and limit long-term professional development (10).

Notably, in certain social environments that emphasize “image management,” appearance-related pressure may become institutionalized. For example, explicit corporate “image requirements” or service-sector norms such as “service with a smile” may further intensify employees’ appearance anxiety. Such structural pressures may be particularly pronounced in collectivist cultures, where individuals face greater difficulty in contesting organizational expectations and norms (22).

### 6.3 Economic burden

Appearance anxiety is also associated with substantial economic costs, encompassing both direct and indirect components. Direct costs include expenditures on cosmetics, skincare products, fitness programs, and medical aesthetic procedures (29). Indirect costs arise from reduced work efficiency due to anxiety, increased healthcare utilization, and opportunity costs such as foregone educational or career opportunities (1).

In some societies, the expansion of the so-called “beauty economy” has normalized appearance-related investment as a rational individual choice. However, such cost–benefit calculations often overlook the accompanying psychological burden, including persistent self-surveillance, continuous pressure to “optimize” appearance, and the risks of investment failures (e.g., unsuccessful cosmetic procedures) (14).

## 7 Limitations and future directions

### 7.1 Methodological limitations

Existing research on appearance anxiety is constrained by several substantial methodological limitations. First, there is an over-reliance on self-report measures, with limited incorporation of behavioral and physiological indicators, resulting in heavy dependence on subjective self-reports (66). Second, the predominance of cross-sectional designs precludes robust causal inference (65). Third, the WEIRD bias (Western, Educated, Industrialized, Rich, Democratic) in sampling practices restricts the generalizability of findings and risks producing a distorted representation of global psychological patterns (68).

In addition, experimental research has largely relied on laboratory-based settings, which offer limited ecological validity (13). Real-world experiences of appearance anxiety are likely shaped by more complex situational factors and coping strategies. Future studies should therefore incorporate ecological momentary assessment and diary-based methodologies to capture the dynamic processes unfolding in everyday life (64).

### 7.2 Need for theoretical integration

Despite a growing body of empirical work, research on appearance anxiety lacks an integrative theoretical framework. Investigations across psychology, sociology, anthropology, and neuroscience remain relatively siloed, constraining a comprehensive understanding of the phenomenon (15). There is a pressing need for interdisciplinary models that integrate micro-level processes (individual cognition and emotion), meso-level dynamics (interpersonal interaction), and macro-level forces (sociocultural structures).

Particular attention should be directed toward the role of power and inequality in shaping appearance anxiety. In social contexts characterized by unequal resource distribution and limited class mobility, appearance may function as one of the few perceived “controllable” forms of capital. Understanding how such structural pressures are translated into individual psychological distress warrants deeper theoretical examination (21).

### 7.3 Directions for improving intervention research

Future intervention research would benefit from longer follow-up periods and more comprehensive outcome assessments (62). Beyond symptom reduction, greater emphasis should be placed on indicators such as quality of life, social functioning, and subjective well-being. Advancing individualized interventions also requires the identification of reliable predictors of treatment response, enabling more precise matching between individuals and therapeutic approaches (55).

Moreover, the development of culturally adaptive interventions is increasingly urgent (68). Simple linguistic translation of Western-based protocols fails to account for culturally specific factors. Instead, intervention models grounded in local cultural resources should be actively developed. At the same time, feasibility across different social systems must be considered, as delivering effective mental health services in settings marked by limited resources and shortages of trained professionals remains a major challenge (50).

### 7.4 Research at policy and social levels

Future research should place greater emphasis on interventions at the policy and societal levels. Potential strategies include media regulation (e.g., restrictions on excessive image retouching), educational reforms (e.g., integrating body positivity education into curricula), and labor protections (e.g., prohibiting appearance-based employment discrimination) (69). Such structural interventions may complement individual-level approaches by addressing broader environmental factors associated with appearance anxiety.

Future research should further examine how broader socioeconomic and institutional contexts influence the development and expression of appearance anxiety. Factors such as media environments, consumer culture, educational systems, and social norms may shape appearance-related experiences in different populations, although the mechanisms involved remain insufficiently understood (16).

## 8 Conclusion and prospects

Appearance anxiety is increasingly recognized as a multidimensional psychological concern with important public mental health implications, arising from the interaction of sociocultural, interpersonal, and individual factors. The evidence reviewed in this article suggests that digital media environments, social comparison processes, and culturally shaped appearance norms may contribute to appearance-related distress, while family relationships, peer interactions, attachment patterns, perfectionism, self-efficacy, and emotion regulation influence individual vulnerability. Importantly, appearance anxiety should not be understood as the result of any single factor but rather as a dynamic process shaped by the continuous interaction between individuals and their social environments.

Current intervention approaches demonstrate varying levels of empirical support. Among the interventions reviewed, cognitive behavioral therapy and its variants possess the strongest overall evidence base. However, much of this evidence originates from studies of related body image disorders, obsessive-compulsive disorder, or other clinically relevant populations rather than appearance anxiety specifically. Consequently, further appearance anxiety-specific intervention studies are needed to establish the generalizability of these findings.

Future research should prioritize longitudinal, experimental, and implementation-oriented designs to clarify causal mechanisms and improve the translation of evidence into practice. Greater attention should also be given to cultural adaptation, measurement validity across populations, and the integration of psychological, interpersonal, and sociocultural perspectives. Advancing the understanding and prevention of appearance anxiety will require interdisciplinary collaboration across clinical psychology, public health, education, and social sciences. Such efforts may contribute to the development of more effective, equitable, and culturally responsive strategies for addressing appearance-related distress in diverse populations.
